# Low-Carbon, Low-Shrinkage Concrete Design Based on Paste–Aggregate Binary Model

**DOI:** 10.3390/ma18143292

**Published:** 2025-07-12

**Authors:** Chunming Lian, Xiong Zhang, Lu Han, Weijun Wen, Wenbiao Lin, Lifang Han

**Affiliations:** 1 Key Laboratory of Advanced Civil Engineering Materials of Education Ministry, School of Material Science and Technology, Tongji University, 4800 Cao’an Road, Shanghai 201804, China; zhangxiong@tongji.edu.cn; 2 China Construction Eighth Bureau Science and Technology Construction Co., Ltd., 899 Gaoke West Road, Shanghai 201804, China; wenweijun@cscec.com (W.W.); linwenbiao@cscec.com (W.L.); hanlifang0810@126.com (L.H.)

**Keywords:** low-carbon concrete, shrinkage control, paste–aggregate binary model, concrete mix design

## Abstract

This study presents a performance-based concrete mix design methodology rooted in the paste–aggregate binary framework, aiming to reduce binder content while ensuring optimal workability and strength. We found that inter-particle spacing (SPT) and paste rheology jointly govern fresh concrete behavior, with slump increasing nonlinearly with SPT and a critical transition zone around 20–35 µm; paste yield stress controls slump, while plastic viscosity governs segregation resistance. A two-level strength model was developed to predict concrete strength from paste properties based on compactness and hydration (R^2^ = 0.90). Fixing SPT at 25 µm was identified as optimal for achieving balanced flowability with minimal paste volume. This approach effectively decouples aggregate packing optimization from paste calibration, offering a physically interpretable and practical framework for designing sustainable, low-carbon, and low-shrinkage concrete.

## 1. Introduction

Concrete remains the indispensable backbone of global infrastructure, but its environmental burden and susceptibility to early-age cracking have become increasingly difficult to ignore [1,2]. Portland-cement manufacture alone accounts for 6~7% of anthropogenic CO_2_ emissions [3], and every additional kilogram of binder added to a mix not only enlarges that footprint but also raises hydration heat [4], autogenous deformation, and drying shrinkage [5].

Paradoxically, most contemporary mix-design prescriptions still favor generous paste volumes to guarantee pumpability and finish ability—particularly since ready-mixed, long-haul, and self-compacting concretes became routine. The Chinese code JGJ 55-2011, for instance, was compiled from empirical water-demand charts more than a decade ago; since then, crushed rock, manufactured sand [6,7], and various solid-waste aggregates [8] with markedly different shapes and gradations have replaced the rounded river gravels on which those charts were based [9]. On site, engineers, therefore, rely on a baseline-mix + trial procedure: they start from tabulated water and sand contents and then adjust water, superplasticizer, and cement up or down until the required slump is attained—usually erring on the side of over-cementing to avoid blockage or segregation [10].

The consequences are evident in a recent survey of 900 production batches across China. For nominal C40 concrete, cement content varied by >180 kg/m^3^, paste volume by >100 L/m^3^, and sand fraction by almost 17 percentage points; similar spreads were observed for C30 and C50 mixtures. Such scatter translates into avoidable emissions (≈150 kg CO_2_ m^−3^ at C40), higher heat release, larger shrinkage, and inconsistent durability performance [11]. A more rational framework is clearly required—one that limits paste to the amount strictly needed for lubrication while preserving workability and strength [12].

Significant research efforts have been dedicated to addressing the environmental impact and cracking susceptibility of concrete. Low-carbon concrete design has emerged as a critical field, focusing on reducing the embodied carbon of cementitious materials through various strategies, including the optimization of aggregate packing, the use of supplementary cementitious materials (SCMs) like fly ash, slag, and calcined clays, and the development of alternative binders [13,14]. These approaches aim to reduce the clinker content and overall cement demand, thereby lowering CO_2_ emissions and energy consumption associated with concrete production. Concurrently, low-shrinkage concrete design has gained prominence, with extensive literature exploring mechanisms of volume change and strategies for its mitigation. These include the judicious selection of aggregates, optimization of mixture proportions to reduce water content, incorporation of shrinkage-reducing admixtures (SRAs), and internal curing techniques [15,16]. While both fields offer promising solutions, a unified approach that simultaneously optimizes both objectives through fundamental material understanding remains less explored.

Recent advances in rheology and packing theory provide the scientific tools to meet this need [17]. When fresh concrete is viewed through a paste–aggregate two-phase lens, it is a concentrated suspension whose flow is governed by cement-paste yield stress (τ_0_) and plastic viscosity (η), both strongly dependent on water-to-binder ratio, supplementary cementitious materials (SCMs) and high-range water-reducing admixtures (HRWRs) [18,19]. In this study, the term “paste–aggregate binary structure” refers to a simplified two-phase representation of fresh concrete, wherein the aggregate particles are modeled as a packed solid skeleton and the cement paste as the continuous suspending medium. This binary approximation excludes interfacial transition zones and air voids from explicit modeling and instead focuses on the geometric and rheological interactions between the paste and aggregate phases.

Paste–aggregate models have evolved significantly in understanding the complex interactions between these phases, particularly concerning particle packing and inter-particle spacing. Statistical packing models—beginning with Andreasen [20], refined by Dinger and Funk [21], and generalized in the Compressible Packing Model (CPM) [22]—show that particle-size distributions following specific power-law exponents maximize aggregate density and thus minimize required paste [23]. These strands converge on a simple insight: it is particle spacing, not abundant paste, that governs workability [24]. If aggregate gradation, shape, and surface area are optimized to keep spacing above a critical limit, the paste layer can be sharply reduced; its rheological “window” (τ_0_ high enough to prevent segregation, η low enough to allow pumping) can then be defined analytically from suspension theory [25].

Despite these theoretical advancements, three barriers have so far limited practical adoption. First, the quantitative coupling of aggregate void structure with the rheological window has not been fully established for real, angular multimodal aggregates [26]. Second, existing packing models rarely incorporate image-based shape descriptors or field-measurable gradation tolerances, making them awkward to apply to regionally variable sands and stones [27]. Third, low-carbon design studies typically tackle CO_2_ reduction and shrinkage mitigation separately, overlooking that paste minimization addresses both simultaneously [28].

The present work closes these gaps by developing a physics-based, low-carbon, low-shrinkage mix-design method anchored in the paste–aggregate model [29]. Our originality lies in several key areas: (1) We establish a quantitative coupling of aggregate void structure with the rheological window specifically for real, angular multimodal aggregates, moving beyond simplified theoretical assumptions. (2) Our method innovatively incorporates image-based shape descriptors and field-measurable gradation tolerances into an adaptive packing curve, thereby enhancing applicability to diverse regional aggregates and eliminating labor-intensive dry-packing trials [30]. (3) Crucially, we present a unified framework that simultaneously addresses CO_2_ reduction and shrinkage mitigation through paste minimization, demonstrating their interconnectedness. On the paste side, a modified Krieger–Dougherty expression embeds the effects of adsorbed and floc-water [31], allowing τ_0_ and η to be predicted directly from grain statistics and HRWR dosage. Linking these sub-models through a surface-area-based paste-thickness index yields the minimum paste volume that still satisfies the rheological window for a given packing. Laboratory validation on 65 paste formulations and 40 full-scale C30–C50 concretes demonstrates cement savings of 8–15%, 28-day shrinkage reductions of up to 25%, and proportional cuts in embodied CO_2_—without sacrificing pumpability or strength.

By unifying aggregate packing, paste rheology, and sustainability objectives into a transparent calculation procedure, this study offers a robust alternative to trial-and-error design and contributes a timely tool for carbon-aware, durability-oriented concrete engineers.

## 2. Theoretical Framework

Concrete, in its fresh state, can be conceptualized as a paste–aggregate binary suspension, where solid aggregate particles are suspended within a continuous cement paste matrix. This fundamental model simplifies the complex multi-phase nature of concrete by focusing on the crucial interactions between these two primary components. Our study anchors its mix design methodology in this binary framework, recognizing that the geometric arrangement of aggregates and the rheological properties of the surrounding paste are paramount to fresh and hardened concrete performance. This approach allows for a decoupled optimization strategy, addressing aggregate packing and paste characteristics independently to achieve desired overall properties.

### 2.1. Aggregate Packing and Particle Spacing

To quantify aggregate packing density, the CPM provides a physically grounded and widely validated framework that considers the interactions among different particle size fractions. When the *i*-th size class dominates the packing structure, its effective packing density $\beta_{i}$ can be calculated as

(1) $$\frac{\beta_{i}}{\gamma_{i}}=1-\sum_{j=1}^{i-1}\left(1-\beta_{i}+b_{ij}\frac{\beta_{i}}{1-\frac{1}{\beta_{i}}}\right)y_{i}-\sum_{j=i+1}^{n}\left(1-a_{ij}\frac{\beta_{i}}{\beta_{j}}\right)y_{j}$$

where $\gamma_{i}$ is the loosest packing density of the i-th fraction, $y_{j}$ is the volume fraction of the j-th size class, and $a_{ij}$ and $b_{ij}$ are empirical coefficients representing the loosening and wall effects, respectively.

(2) $$a_{ij}=\sqrt{1-{\left(1-\frac{d_{j}}{d_{i}}\right)}^{1.02}}$$

(3) $$b_{ij}=1-{\left(1-\frac{d_{i}}{d_{j}}\right)}^{1.50}$$

The empirical coefficients $a_{ij}$ and $b_{ij}$, summarized and established by Professor Larrard based on extensive experimental data [22], are assessed through a rigorous experimental calibration procedure. As illustrated in Equations (2) and (3), these coefficients are precisely determined by employing numerical optimization algorithms to fit the model’s predictions to experimentally measured aggregate packing densities. This ensures the accurate characterization of the specific packing behavior of the aggregate system under investigation and aligns with Larrard’s classic work.

The overall packing density Φ of t the blended aggregate system is then computed by introducing a compaction index K, which accounts for the compaction method (typically, K = 8 for pumped concrete):

(4) $$K=\sum_{i=1}^{n}\frac{y_{i}/\beta_{i}}{1/\Phi -1/\gamma_{i}}$$

The actual packing density is obtained by directly solving this system of equations. A key geometric indicator of fresh-state behavior is the inter-particle spacing (SPT, μm), representing the average distance between aggregate surfaces. It is calculated by

(5) $$SPT=\frac{\phi_{max}-\phi }{S_{v}\phi_{max}\phi }$$

where ϕ is the aggregate volume fraction, and $S_{v}$ is the specific surface area of the aggregate system (m^2^/m^3^). Unlike idealized models, real aggregates are irregular and polydisperse. A modified expression for $S_{v}$ incorporates both particle size distribution and shape:

(6) $$S_{v}=\frac{{27P}_{s}}{{28A}_{s}}\sqrt{1+{\left(\frac{D_{max}}{D_{min}}\right)}^{2}}$$

Here, $P_{s}$, $A_{s}$, $D_{max}$, and $D_{min}$ are all obtained from image analysis. Specifically, $P_{s}$ represents the average perimeter of aggregate particles, $A_{s}$ is the average projected area, while $D_{max}$ and $D_{min}$ denote the major and minor axes of the particle’s fitted ellipse, respectively. The term $\sqrt{1+{\left(D_{max}/D_{min}\right)}^{2}}$ quantifies the broadness of particle morphology and shape anisotropy.

The aggregate shape descriptors, including perimeter ($P_{s}$), area ($A_{s}$), maximum Feret diameter ($D_{max}$), and minimum Feret diameter ($D_{min}$), were extracted via 2D digital image analysis of representative samples. While 3D techniques such as X-ray computed tomography provide detailed volumetric information, they are typically applied to limited sample sizes and cannot fully represent the morphology of the entire aggregate population. In contrast, 2D analysis offers significantly higher efficiency and, based on stereological principles, can effectively reflect particle roughness and surface texture.

Although the accuracy of 2D measurements may be influenced by image resolution and processing methods (e.g., thresholding and edge detection), a standardized protocol was strictly followed to ensure consistency and comparability. While 2D projections cannot reconstruct full 3D geometry, the derived parameters provide reliable input for relative comparisons and predictive modeling within the defined methodological framework.

### 2.2. Rheological Design for Paste–Aggregate Suspensions

In the paste–aggregate binary framework, concrete rheology is determined by the interaction between the packed aggregate skeleton and the surrounding paste. A two-step mix design strategy has been adopted:

Step 1: Aggregate Skeleton Optimization

The aggregate system is optimized using CPM to maximize packing density while targeting a desired SPT (typically 20–40 μm). This ensures efficient lubrication without excessive paste content. The required aggregate volume fraction ϕ is derived from

(7) $$\phi =\frac{\phi_{max}}{S_{v}\phi_{max}SPT+1}$$

This equation is derived from the definition of SPT and expresses the minimum aggregate volume fraction needed to maintain a specified paste layer thickness, building on stereological theory and image-based surface area estimation [27].

Step 2: Paste Rheology Adjustment

Once the aggregate structure is fixed, the paste is tailored to meet specific workability and stability requirements. The yield stress $\tau_{p}$ (Pa) governs flow initiation (slump), while the plastic viscosity $\mu_{p}$ (Pa·s) controls segregation resistance.

The rheological behavior of concrete is related to that of the paste by

(8) $$\tau_{c}=\tau_{p}\sqrt{\frac{1-\phi }{{\left(1-\phi /\phi_{max}\right)}^{4.7\phi_{max}}}}$$

(9) $$\mu_{p}\geq \frac{\left(\rho_{g}-\rho_{p}\right)g{T_{f}D}^{2}}{18\;SPT}$$

where $\rho_{g}$ and $\rho_{p}$ are the densities of aggregates and paste, respectively, and $T_{f}$, D represent time and particle size scales relevant to flow stability. These expressions are adapted from suspension rheology and Stokesian stability theory, capturing the influence of solid fraction and spacing on yield behavior and segregation resistance [32,33].

This decoupled approach enables the independent optimization of aggregate structure and paste properties, resulting in a robust and performance-oriented mix design methodology.

### 2.3. Strength Model of Paste–Aggregate Structured Concrete

In paste–aggregate binary structured concrete, compressive strength is predominantly governed by the hardened paste, which serves as the continuous load-bearing phase. Aggregates primarily function as inert fillers, providing dimensional stability and mechanical confinement, but contribute minimally to the intrinsic strength of the composite. Consequently, strength modeling efforts are focused on the paste phase, with its mechanical performance determined by compositional parameters and the degree of hydration.

A two-level semi-empirical strength model is proposed:

Level 1: Paste Strength Prediction

The compressive strength of hardened paste $f_{cp}$, is estimated as

(10) $$f_{cp}=34.7\alpha \left(t_{e}\right)R_{c_{28}}{\left[\frac{\rho_{p}}{\rho_{c}\left(1+{w/c}^{\prime }\right)}\right]}^{3.1}$$

where

- $\alpha \left(t_{e}\right)$ is the equivalent hydration degree at curing age $t_{e}$;
- $R_{c_{28}}$ is the 28-day cement strength, MPa;
- $\rho_{p}$ and $\rho_{c}$ are the apparent and cement paste densities, kg/m^3^;
- ${w/c}^{\prime }$ is the effective water–cement ratio, corrected for the presence of mineral admixtures.

This formulation captures the role of hydration, binder density, and dilution in determining paste strength.

Level 2: Concrete Strength Estimation

Equation (10) is based on a compactness–hydration strength model that incorporates the effects of binder dilution and hydration degree, while Equation (11) maps paste strength to concrete strength via a nonlinear saturation function reflecting confinement and ITZ effects. The overall concrete strength $f_{c}$ is expressed as

(11) $$f_{c}=\frac{p_{1}f_{cp}}{p_{2}+f_{cp}}$$

where $p_{1}$ and $p_{2}$ are empirical constants reflecting microstructural efficiency and saturation effects. This function accounts for diminishing strength returns at high paste strength due to stress localization and cracking.

The framework presented in this section integrates aggregate packing theory with paste rheology and hydration-based strength modeling. The use of inter-particle spacing (SPT) as a control parameter offers a physically interpretable basis for optimizing flowability, segregation resistance, and strength. This approach is especially valuable for concretes using low binder content or blended cements, where traditional models based solely on the water–cement ratio are inadequate.

## 3. Materials and Experimental Methods

### 3.1. Cementitious Materials

The cement used in this study was ordinary Portland cement (P·O 42.5), complying with the Chinese standard GB 175 [34]. The physical properties of the cement are summarized in Table 1.

Densities were measured using a Le Chatelier flask and kerosene as per Chinese standard GB/T 208 [35]. Particle size distributions were obtained via laser diffraction analysis. See Table 2.

For this study, Portland cement and these SCMs were combined to form the total cementitious binder. The specific influence and individual proportions of FA, GGBS, and LP are not the primary focus of this paper; rather, the blended cementitious material is treated as a unified binder system whose combined properties dictate the overall paste performance. This approach is consistent with considering a blended binder as essentially a different “grade” of cement, simplifying the mix design problem to optimizing paste volume based on the characteristics of the given binder system. A detailed investigation into the individual effects and optimal proportions of different SCMs will be explored in future research.

### 3.2. Coarse Aggregates and Sand

**Figure 1:**
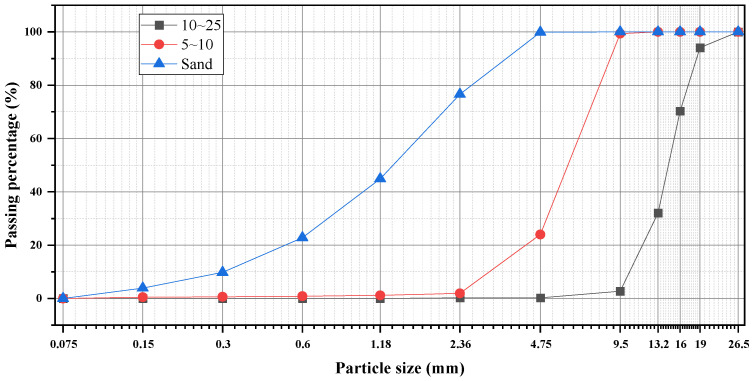
Aggregate gradation curves.

Two sizes of coarse aggregates (5–10 mm and 10–20 mm) and manufactured sand were used in varying ratios. Aggregate packing densities were measured to support the model validation. The particle size distributions of all aggregates are shown in Figure 1.

The aggregates employed in this study were locally sourced, high-quality crushed limestone. The coarse aggregates exhibited an angular to sub-angular shape with a rough surface texture, typical of crushed rock. Their apparent density was measured as 2680 kg/m^3^ for both sizes, with a water absorption rate of approximately 0.8% by mass. The manufactured sand also demonstrated an angular morphology and a rough surface texture, with an apparent density of 2650 kg/m^3^, a water absorption rate of 1.2%, and a fineness modulus of 2.95. The primary mineralogical composition of all aggregates was calcium carbonate.

### 3.3. Experimental Design

Three experimental programs were developed based on the paste–aggregate binary framework:

(1) Influence of Aggregate Volume Fraction on Workability

The paste composition and aggregate gradation were fixed, while aggregate volume fraction was varied (62–78%) to control the inter-particle spacing (SPT). Mix details are shown in Table 3. For mixtures with SPT < 0, the paste volume was insufficient to fill the voids between aggregates.

(2) Effect of Paste Rheology on Concrete Workability

Paste volume and aggregate gradation were fixed. Paste rheology was modified by adjusting the water–binder ratio and superplasticizer dosage. Yield stress ranged from 0.34–4.18 Pa, and plastic viscosity ranged from 11.95–100.29 Pa·s (Table 4).

(3) Strength Verification of the Paste–Aggregate Model

Concrete mixes targeting C30, C40, and C50 strength grades were prepared (Table 5). Within each grade, the water–cement ratio ranged from 0.32 to 0.44 and the sand ratio from 36% to 44%. Compressive strength at 7 and 28 days and slump were recorded.

### 3.4. Test Methods

Workability and strength were assessed through standardized laboratory tests:

(1) Paste Rheology: Measured using a coaxial cylinder rotational rheometer; Bingham model fitting provided yield stress and plastic viscosity.
(2) Concrete Slump: Determined using standard slump cone per GB/T 50080-2016 [36] (ASTM C143 [37]).
(3) Slump Flow: Measured as per EFNARC and GB/T 50080-2016; V-funnel time recorded to evaluate flow time.
(4) Compressive Strength: 150 mm cubes tested at 7 and 28 days per GB/T 50081-2019 [38] (ASTM C39 [39]); an average of three specimens reported.

All specimens were cured under standard conditions (20 ± 2 °C, RH, relative humidity ≥ 95%) unless stated otherwise.

## 4. Results and Discussion

### 4.1. Key Parameters Governing Workability: Particle Spacing and Paste Rheology

The workability of fresh concrete, fundamentally a concentrated suspension, is primarily governed by the intricate interplay between aggregate packing density (dictating inter-particle spacing) and the rheological properties of the surrounding cement paste. To investigate this, concrete mixes with identical paste composition and aggregate grading were tested while varying aggregate volume fraction to control inter-particle spacing (SPT).

**Figure 2:**
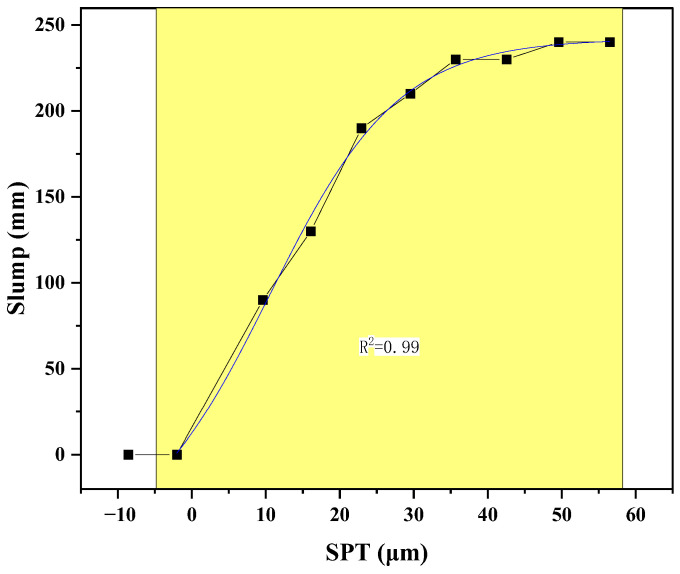
Influence of inter-particle spacing on workability.

As shown in Figure 2, slump exhibited a clear sigmoidal response to SPT, a trend highly consistent with observations in other particle-filled suspensions and granular materials, such as those reported by Ferraris et al. [40] and Roussel et al. [41]. Paste was demonstrably insufficient to fill aggregate voids, resulting in a jammed, solid-like structure with near-zero slump. This corresponds to a microstructural condition where aggregate particles are in direct, continuous contact, preventing macroscopic flow due to high inter-particle friction and mechanical interlocking. As SPT increased beyond approximately 20 μm, slump rose sharply, indicating the formation of a sufficient lubricating paste layer between particles. This “transition zone” (between 20 and 35 μm) signifies the critical shift from a jammed state to a flowable suspension, where the paste begins to effectively separate aggregates and facilitate their relative movement, overcoming inter-particle friction. This behavior aligns with the concept of a percolation threshold for the paste phase, where a continuous path of lubricating paste allows bulk flow [42].

When SPT exceeded 40 μm, slump plateaued, suggesting a fully lubricated regime where further increases in paste film thickness yielded diminishing returns in flowability (Figure 2). In this regime, the overall workability was governed primarily by the intrinsic rheological properties of the paste itself, rather than the geometric constraints of particle packing.

An SPT value less than zero corresponds to a theoretical underfilling condition, where the available paste volume is insufficient to fill the voids between aggregate particles. This results in a jammed state with no effective lubrication between particles, leading to near-zero flowability. While SPT itself is a derived geometric parameter rather than a directly measurable physical quantity, negative values analytically indicate a non-flowable mixture and help distinguish underfilled systems from lubricated ones.

The relationship between slump and particle spacing (SPT) was successfully fitted using a Boltzmann sigmoid function, achieving an excellent coefficient of determination (R^2^ = 0.99). The fitted equation is expressed as

(12) $$Slump=\frac{A_{1}-A_{2}}{1+exp\left(\frac{SPT-x_{0}}{dx}\right)}+A_{2}$$

The physical meanings of the fitting parameters are as Table 6 shows:

The excellent fit demonstrates that the Boltzmann function effectively characterizes the nonlinear transition between jammed and lubricated states in concrete, confirming previous conceptual models for paste–aggregate systems [43]. These results underscore that inter-particle spacing is a key geometric parameter controlling concrete workability within the paste–aggregate binary framework.

It is important to emphasize that while the specific parameters of this Boltzmann fit are unique to the materials tested, the sigmoidal relationship itself reflects a fundamental physical transition common to concentrated suspensions. The strength of the paste–aggregate binary framework is its generalizability; by experimentally determining the key input parameters for any new set of materials—namely, the aggregate packing characteristics (ϕ, $S_{v}$) and the paste rheology ($\tau_{p}$ $\mu_{p}$)—the methodology can be applied to predict and design the workability of a wide variety of concrete mixtures.

**Figure 3:**
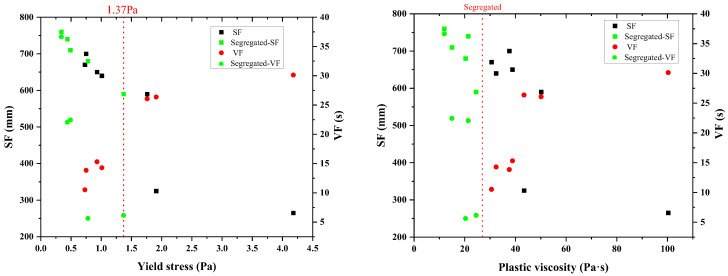
Influence of paste rheology on concrete segregation resistance.

Beyond sufficient lubrication, paste rheology (specifically yield stress and plastic viscosity) plays a critical role in controlling fresh concrete behavior, particularly segregation resistance. As shown in Figure 3, mixtures incorporating paste with low yield stress exhibited relatively high flowability but were prone to segregation. However, the occurrence of segregation could not be eliminated solely by increasing yield stress. Notably, when the plastic viscosity of the paste fell below a certain threshold (e.g., <25 Pa·s in this study), visible segregation was observed, even in mixtures with moderate yield stress. This observation aligns with established principles of suspension stability, where sufficient inter-particle attractive forces and matrix viscosity are required to counteract gravitational settlement of larger particles [44].

This finding suggests that plastic viscosity, rather than solely yield stress, is the dominant rheological parameter governing segregation resistance in the range tested. Higher viscosity improves the suspension stability of the system by providing greater resistance to aggregate migration and particle sedimentation. This is because a higher viscosity paste can better suspend larger aggregate particles, preventing them from settling or moving apart under their own weight or during placement. However, if viscosity becomes too high (e.g., >80 Pa·s), flowability declines sharply, making the mixture impractical for placement, highlighting the need for a balanced rheological profile. For a given aggregate structure, there exists a critical lower bound of plastic viscosity below which segregation becomes unavoidable, indicating a threshold for stable suspension. To ensure both sufficient flowability and adequate stability, paste composition must be optimized to maintain plastic viscosity within an acceptable range.

### 4.2. Strength Dependency on Paste Properties and Predictive Model Validation

In the paste–aggregate binary framework, the compressive strength of hardened concrete is predominantly governed by the mechanical properties of the continuous cement paste matrix, which acts as the primary load-bearing phase. Aggregates primarily provide volumetric stability and contribute to the composite’s overall stiffness, with their strength becoming critical only at very high paste strengths or specific loading conditions.

To validate the proposed strength model, ten concrete mixtures with fixed aggregate structure and varying paste compositions were prepared. The compressive strength of the paste phase ($f_{cp}$) was calculated using the hydration-informed compactness model (Equation (13)) described in Section 2.3, which incorporates the water-to-binder ratio, hydration degree, and paste density. Both 7-day and 28-day strengths were considered to reflect the evolution of mechanical properties.

**Figure 4:**
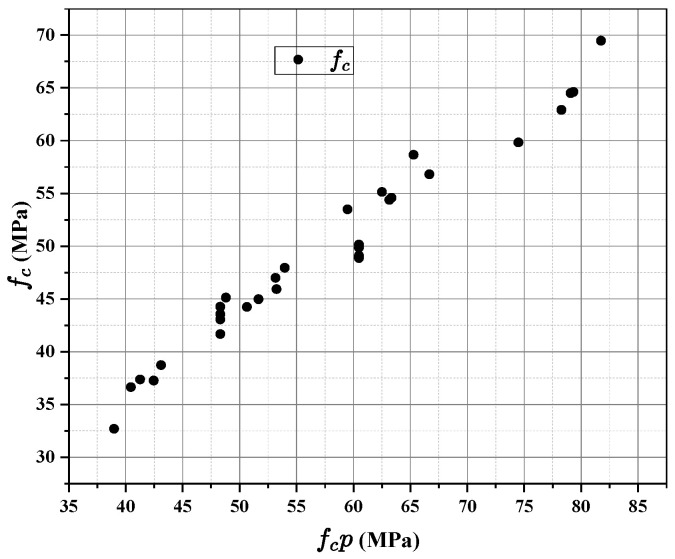
Correlation between slurry strength and concrete strength.

As shown in Figure 4, the measured concrete compressive strength ($f_{c}$) was plotted against the calculated paste strength. A clear non-linear relationship was observed, and the data were accurately fitted using a rectangular hyperbola (R^2^ = 0.90), consistent with numerous empirical models describing the composite strength of concrete, such as those proposed by Popovics [45] and Kaplan [46]. The fitted equation is

(13) $$f_{c}=\frac{p_{1}f_{cp}}{p_{2}+f_{cp}}$$

This empirical model reflects the composite nature of concrete, where paste strength dominates the early stage (paste-controlled), transitions through an interfacial zone (ITZ-controlled), and eventually saturates due to aggregate constraints (aggregate-controlled).

At low $f_{cp}$ (e.g., $f_{cp}$ < 30 MPa), the concrete strength increases nearly linearly, suggesting that paste quality (dictated by the water-to-binder ratio and hydration degree, reflecting the density and integrity of the hydrated cement matrix and its hydration products) is the limiting factor. In this regime, failure typically occurs through the bulk paste or the weak portions of the interfacial transition zone (ITZ).

Near $f_{cp}$ = 50 MPa, the strength gain rate decreases, indicating that the Interfacial Transition Zone (ITZ) becomes the dominant failure zone. The ITZ, a region of higher porosity and lower density around aggregate particles formed due to wall effect and preferential water accumulation during mixing, becomes the weakest link as the bulk paste strengthens. This observation is well documented in concrete science [47].

At high $f_{cp}$ > 70 MPa, the concrete strength approaches an upper limit, beyond which further strengthening of the paste yields diminishing returns. In this regime, failure is often initiated by aggregate crushing or bond failure at the aggregate–paste interface, indicating that the aggregate strength and the mechanical interlocking within the skeleton become the limiting factors, as commonly observed in high-strength concrete [48].

**Figure 5:**
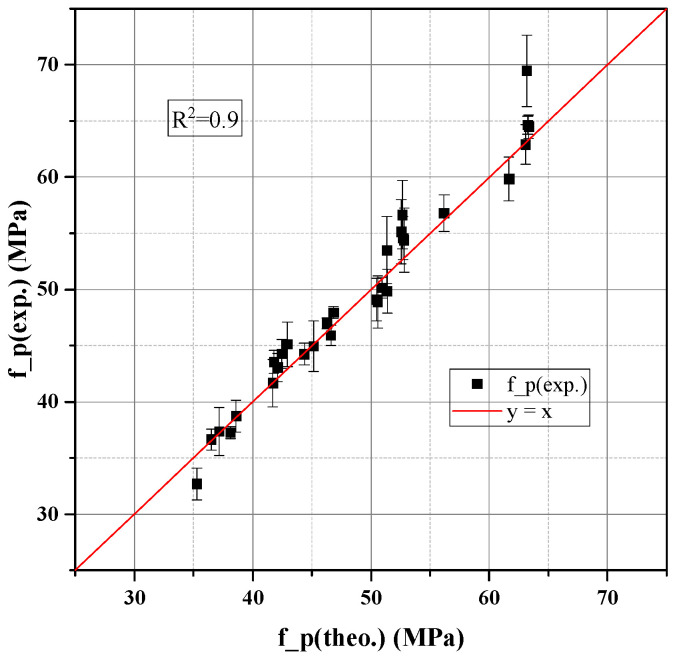
Prediction accuracy of concrete strength model.

The best-fit parameters were as follows (Figure 5):

$p_{1}$ = 363.5 MPa (maximum achievable concrete strength);

$p_{2}$ = 324.1 MPa (critical paste strength corresponding to half-saturation);

Coefficient of determination R^2^ = 0.90.

These results robustly confirm that concrete strength is predominantly governed by the paste phase when the aggregate skeleton is fixed, validating the core assumption of the paste–aggregate binary framework. The proposed model not only captures the nonlinear strength development but also enables strength prediction based solely on paste properties, providing a robust tool for performance-based mix design with mechanistic underpinnings. The high R^2^ value compares favorably with other predictive models in the literature for similar material systems.

### 4.3. Toward Low-Carbon and Low-Shrinkage Concrete Mix Design

Leveraging the insights gained from understanding the roles of particle spacing, paste rheology, and paste-controlled strength, a four-step design methodology was developed within the paste–aggregate binary framework. This approach systematically minimizes paste volume while ensuring adequate workability, strength, and segregation resistance, directly contributing to the design of sustainable, low-carbon, and low-shrinkage concrete.

Step 1: Aggregate Optimization under Fixed Paste Layer Thickness

The first step involves characterizing the aggregate skeleton. The packing void ratio ($\phi_{viod}$) and specific surface area ($S_{v}$) of the selected aggregate system are experimentally measured using the dry-rodded packing method and laser particle analysis, respectively. A target paste layer thickness (e.g., 25 μm) is then selected to ensure lubrication and minimize particle contact.

Based on packing theory, the required aggregate volume fraction (ϕ) is calculated from

(14) $$\phi =\frac{\phi_{max}}{S_{v}\phi_{max}SPT+1}$$

The aggregate gradation is iteratively optimized (using the Compressible Packing Model, CPM) to $\phi_{max}$ and hence minimize the required paste volume, which directly contributes to carbon reduction and shrinkage control by lowering cement content.

Step 2: Yield Stress Inversion from Target Slump

Given a target slump value (e.g., 180 mm) for the concrete mixture, the required concrete yield stress ($\tau_{c}$) is estimated using empirical correlations or inverse models developed in Section 4.1. Under a fixed aggregate structure, $\tau_{c}$ can be related to paste yield stress ($\tau_{p}$) through a suspension model:

(15) $$\tau_{c}=\tau_{p}\sqrt{\frac{1-\phi }{{\left(1-\phi /\phi_{max}\right)}^{4.7\phi_{max}}}}$$

This relation allows the determination of the target paste yield stress that ensures the desired workability at minimum volume.

Step 3: Strength-Oriented Initial Paste Mix Design

Based on the required mechanical performance, the paste compressive strength $f_{cp}$ is predicted using the hydration-based compactness model (Equation (13)), and the corresponding concrete strength is validated via the macro-to-micro strength transformation (Equation (14)). Reverse-engineering these models enables the estimation of the effective water-to-binder ratio (w/b)’, binder composition, and paste density required to achieve the desired strength.

The resulting mix design yields the initial paste proportions that balance flowability and strength under minimal binder content.

Step 4: Segregation Resistance Check and Viscosity Tuning

Finally, the plastic viscosity of the paste is evaluated to ensure adequate resistance to segregation. As observed in Section 4.1, segregation tends to occur when viscosity falls below a critical threshold (e.g., ~25 Pa·s). The paste composition is adjusted—typically by modifying fine filler content, viscosity-enhancing admixtures, or superplasticizer dosage—to achieve

(16) $$\mu_{p}\geq \frac{\left(\rho_{g}-\rho_{p}\right)g{T_{f}D}^{2}}{18SPT}$$

Only when the paste satisfies both yield stress (flowability) and plastic viscosity (stability) requirements is the mix deemed acceptable.

**Figure 6:**
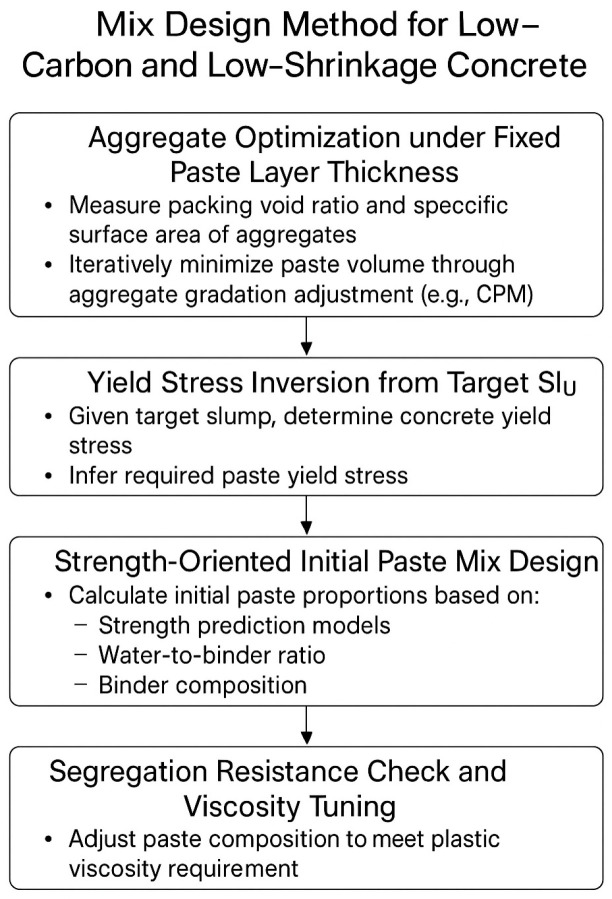
Mix design method for low-carbon and low-shrinkage concrete.

This four-step method systematically integrates aggregate structure optimization, rheological modeling, and strength prediction into a unified framework for designing low-carbon, low-shrinkage concrete (Figure 6). By minimizing paste content while maintaining performance, it provides a practical pathway toward sustainable concrete tailored to project-specific requirements. The “low-carbon” aspect of this methodology primarily refers to the direct reduction in the total cementitious binder content required to achieve a target concrete performance (workability and strength). While a full life-cycle assessment (LCA) that includes all material and construction phases is beyond the scope of this study, the proposed method provides a fundamental and quantifiable pathway for significant CO_2_ emissions reduction at the critical mix design stage. It is important to note that while this methodology provides a robust framework demonstrated within the scope of this study, its broader application and refinement across diverse material types and unexamined variables are discussed as key directions for future research in the Section 5.

The “low carbon” aspect of this methodology primarily refers to the direct reduction in the total cementitious binder content required to achieve a target concrete performance (workability and strength). As the production of cementitious binders is the largest contributor to concrete’s carbon footprint (typically ranging from 0.8 to 0.9 kg CO_2_ equivalent per kg of cementitious material, depending on clinker content and SCMs), minimizing this volume directly translates to lower embodied CO_2_ emissions. For instance, conventional concrete mix designs for C40 strength might typically require total binder contents of 450–500 kg/m^3^. In contrast, our method, by optimizing aggregate packing to reduce void volume and efficiently utilize the paste, enables the design of C40 concrete with a binder content as low as ~400 kg/m^3^ (as exemplified in Table 5 for C40). This represents a significant binder reduction of approximately 50–100 kg/m^3^ per cubic meter of concrete.

Translating this into CO_2_ savings, this can lead to an estimated reduction of 40–85 kg CO_2_ equivalent per cubic meter of concrete. This strategic reduction in binder volume not only lowers the environmental footprint but also inherently mitigates drying shrinkage risk, making it a robust way to design sustainable concrete tailored to specific performance needs. While a full life-cycle assessment (LCA) that includes all material and construction phases is beyond the scope of this study, the proposed method provides a fundamental and quantifiable pathway for significant CO_2_ emissions reduction at the critical mix design stage.

## 5. Conclusions

This study develops a performance-based concrete mix design method grounded in the paste–aggregate binary framework, with the dual objectives of minimizing binder content and ensuring sufficient workability and strength. The following conclusions are drawn:

(1) Inter-particle spacing (SPT) and paste rheology jointly control fresh-state behavior. The experimental results show that slump increases nonlinearly with SPT and plateaus when SPT exceeds 40 µm. A transition zone between 20 and 35 µm marks a sensitive range for flowability. The paste yield stress governs the onset of flow, while plastic viscosity is the key parameter controlling segregation resistance.
(2) Concrete strength is governed primarily by the hardened paste phase. A two-level predictive model was proposed and validated: paste strength is estimated based on the water-to-binder ratio, hydration degree, and density and then mapped to the concrete strength via a hyperbolic transformation. The model exhibits strong agreement with experimental data (R^2^ = 0.90) and offers mechanistic insight into strength development.
(3) Fixing SPT at 25 μm ensures balanced flowability and reduced paste demand. The findings of this study demonstrate that an optimal inter-particle spacing (SPT) of approximately 25 micrometers is indispensable for attaining the requisite workability and pumpability characteristics of the developed paste. This specific SPT value facilitates adequate particle lubrication and homogeneous flow within the matrix. Critically, for a defined aggregate system, the precise optimization of its gradation to achieve a target SPT (e.g., 25 μm) yields both sufficient particle lubrication and robust workability, concurrent with a minimized paste volume requirement. This establishes a fundamental geometric basis for the advancement of sustainable, low-carbon mix designs.
(4) Mix design can be decoupled into aggregate structure control and paste calibration. Once the aggregate packing and SPT are fixed, paste properties are adjusted to meet strength and rheological performance targets. Yield stress is tuned for workability, and plastic viscosity is adjusted—using SCMs or admixtures—to resist segregation.
(5) The proposed method enables the design of low-carbon, low-shrinkage concrete.

This optimized approach, which controls inter-particle spacing, not only boosts material performance but also offers a powerful strategy for creating more sustainable construction materials. By significantly cutting down on high-carbon cement and minimizing binder volume, our method directly lowers the environmental footprint. It decouples strength from excess paste, reducing both cement use and shrinkage risk, making it a robust way to design sustainable concrete tailored to specific performance needs.

This research successfully established a novel and robust theoretical and practical framework for concrete mix design, fundamentally rooted in the principles of particle-scale structural control and paste-level performance optimization. By explicitly and quantifiably linking inter-particle spacing to paste rheology and ultimately to macroscopic mechanical strength, this framework offers an unprecedented capacity for the rational and optimized design of high-performance, resource-efficient, and sustainable concrete mixtures. This approach, especially valuable for concretes using low binder content or blended cements where traditional models based solely on the water–cement ratio are inadequate, represents a significant advancement in the development of next-generation concrete technologies.

While this methodology provides a robust framework demonstrated within the specific scope of this study, it is important to acknowledge that the current experimental validation employed a single type of crushed limestone aggregate and a P.O 42.5 cement blended with specific supplementary cementitious materials. Therefore, the empirical coefficients and practical guidelines derived are inherently tied to this material set, limiting immediate universal generalizability. Furthermore, though our models offer mechanistic insights, a deeper quantitative understanding of how specific microstructural properties of diverse materials precisely influence macro-level rheological and mechanical behavior warrants further investigation. The current methodology for aggregate packing analysis predominantly employs a deterministic approach; however, recognizing the inherent variability in particle arrangement within granular systems, a purely deterministic assessment may only yield approximate results.

Consequently, future research should rigorously incorporate probabilistic functions into this methodology. This advancement is anticipated to enable a more robust accounting for inherent material variability, facilitate the presentation of design outcomes with a defined level of probability, and ultimately foster a more comprehensive and statistically robust understanding of aggregate packing behavior. Future work should also focus on validating and refining the proposed methodology across a wider range of aggregate types (e.g., gravel, different rock types, and recycled aggregates) and diverse cementitious binders (e.g., various SCM combinations, alkali-activated materials, and alternative cements) and under varied environmental conditions. This will be crucial to enhance the framework’s generalizability, further establish its robustness, and explore its applicability in broader engineering contexts, including long-term durability and structural performance.

## Figures and Tables

**Table 1:** Physical properties of P·O 42.5 cement.

Specific Surface Area (m^2^/kg)	Density (kg/m^3^)	Standard Consistency (%)	Initial Setting Time (min)		Final Setting Time (MPa)		Compressive Strength (MPa)	
317	2990	28.4	193	266	5.3(3d)	9.3(28d)	19.3(3d)	43.2(28d)

**Table 2:** Physical properties of cementitious powders.

Material	Apparent Density (g/cm^3^)	Specific Surface Area (m^2^/kg)	Surface Area per Volume (m^2^/m^3^)
Cement (C)	2.99	315	0.94

**Table 3:** Concrete mix proportions with varying aggregate volume fractions.

Mix ID	Cement (kg)	Water (kg)	Sand (kg)	5~10 mm (kg)	10~20 mm (kg)	HRWR (kg)	SPT (μm)	Aggregate Volume Fraction (%)
A-1	265	122	975	415	685	3.6	−8.6	77.67
A-2	287	132	952	405	668	3.9	−2.04	75.8
A-3	324	149	913	388	641	4.4	9.6	72.68
A-4	343	158	892	380	626	4.6	16.08	71.05
A-5	362	167	872	371	612	4.9	22.94	69.41
A-6	380	175	853	363	599	5.1	29.54	67.9
A-7	396	182	836	356	587	5.3	35.64	66.56
A-8	413	190	818	348	574	5.6	42.54	65.1
A-9	430	198	800	340	562	5.8	49.59	63.68
A-10	445	205	783	333	550	6	56.49	62.35

Note: HRWR stands for high-range water-reducing admixtures.

**Table 4:** Concrete workability with varying paste rheological parameters.

Mix ID	Plastic Viscosity (Pa·s)	Yield Stress (Pa)	Slump Flow (mm)	VF (s)	Remarks
B-1	100.29	4.18	265	30.13	
B-2	50.14	1.76	590	26.07	
B-3	38.85	0.93	650	15.31	
B-4	37.59	0.75	700	13.84	
B-5	43.42	1.91	325	26.37	
B-6	32.41	1.01	640	14.28	
B-7	30.57	0.73	670	10.52	
B-8	21.4	0.44	740	22.06	Segregated
B-9	24.51	1.37	590	6.15	Segregated
B-10	20.32	0.78	680	5.63	Segregated
B-11	14.94	0.49	710	22.44	Segregated
B-12	11.95	0.34	760	36.65	Segregated

**Table 5:** Mix proportions and compressive strength of concrete.

Strength Grade	w/c	Sand Ratio (%)	Cement (kg/m^3^)	Sand (kg/m^3^)	Coarse Aggregate (kg/m^3^)	Water (kg/m^3^)	HRWR (%)	Slump (mm)	7d	28d
C30	0.44	36	330	709	1260	145	1.00	140	33	45
		38	330	748	1221	145	1.00	135	37	47
		40	330	788	1181	145	1.00	156	39	48
		42	330	827	1142	145	1.00	141	37	45
		44	330	867	1102	145	1.00	124	37	44
C40	0.36	36	400	686	1221	144	1.00	165	42	49
		38	400	724	1183	144	1.00	171	44	49
		40	400	763	1144	144	1.00	178	44	50
		42	400	801	1106	144	1.00	182	46	57
		44	400	839	1068	144	1.00	168	43	50
C50	0.32	36	470	657	1168	150	1.20	180	53	60
		38	470	694	1131	150	1.20	187	55	63
		40	470	730	1095	150	1.20	193	59	69
		42	470	767	1058	150	1.20	174	55	65
		44	470	803	1022	150	1.20	169	54	65

**Table 6:** Physical meanings of the fitting parameters.

Parameter	Mathematical Role	Physical Interpretation
$A_{1}$	Lower asymptote (minimum slump)	Represents the no-flow regime where the paste volume is insufficient to fill inter-aggregate voids, leading to jamming and zero slump.
$A_{2}$	Upper asymptote (maximum slump)	Denotes the fully lubricated regime, where paste sufficiently separates and lubricates aggregates, achieving maximum flowability.
$x_{0}$	Inflection point	Identifies the critical particle spacing at which the transition from jamming to flowable behavior occurs. This value marks the threshold SPT (~20–30 μm in this study) required to initiate significant flow.
dx	Slope-related parameter	Reflects the width of the transition zone, i.e., how sensitive the concrete workability is to changes in SPT. A small dx indicates a sharp transition, whereas a larger dx implies a more gradual response.

## Data Availability

The original contributions presented in this study are included in the article. Further inquiries can be directed to the corresponding authors.

## Abbreviations

The following abbreviations are used in this manuscript:

SCM	Supplementary Cementitious Materials
SRA	Shrinkage-Reducing Admixtures
HRWR	High-Range Water-Reducing admixtures
CPM	Compressible Packing Model
SPT	Surface area-based Paste Thickness
VF	V-Funnel outflow time
SF	Slump Flow

## References

[1] Carrasquillo M.L.S.a.R.L. (1989). Creep and Shrinkage Properties in Concrete Containing Fly Ash.

[2] Mehta P.K., Burrows R.W. (2001). Building durable structures in the 21st century. Concr. Int..

[3] Schneider M. (2019). The cement industry on the way to a low-carbon future. Cem. Concr. Res..

[4] Committee A.C.I. (1990). Effect of Restraint, Volume Change, and Reinforcement on Cracking of Mass Concrete. Mater. J..

[5] Kwan A.K.H., Ling S.K. (2015). Lowering paste volume of SCC through aggregate proportioning to reduce carbon footprint. Constr. Build. Mater..

[6] Shen W., Yang Z., Cao L., Cao L., Liu Y., Yang H., Lu Z., Bai J. (2016). Characterization of manufactured sand: Particle shape, surface texture and behavior in concrete. Constr.Build. Mater..

[7] Chen X., Yu-guang G., Li B., Zhou M., Li B.-X., Liu Z.A., Zhou J. (2020). Coupled effects of the content and methylene blue value (MBV) of microfines on the performance of manufactured sand concrete. Constr. Build. Mater..

[8] Xuan D., Zhan B., Poon C.s. (2016). Assessment of mechanical properties of concrete incorporating carbonated recycled concrete aggregates. Cem. Concr. Compos..

[9] Yang Y., Chen B., Su Y., Chen Q., Li Z., Guo W., Wang H. (2020). Concrete Mix Design for Completely Recycled Fine Aggregate by Modified Packing Density Method. Materials.

[10] Lv M., Jiao A., An X., Bai H., Zhang J., Shao K. (2022). Development of a Mix Design Method for Multiplexed Powder Self-Compacting Concrete Based on the Multiscale Rheological Threshold Theory. Buildings.

[11] Mohamed A.M., Tayeh B.A., Majeed S.S., Aisheh Y.I.A., Salih M.N.A. (2024). Fresh, hardened, durability and microstructure properties of seawater concrete: A systematic review. J. CO_2_ Util..

[12] Zhang J., An X., Li P. (2020). Research on a mix design method of self-compacting concrete based on a paste rheological threshold theory and a powder equivalence model. Constr. Build. Mater..

[13] Ahmed M.M., Sadoon A., Bassuoni M.T., Ghazy A. (2024). Utilizing Agricultural Residues from Hot and Cold Climates as Sustainable SCMs for Low-Carbon Concrete. Sustainability.

[14] Wang X.-Y., Lee H.-s., Gao X.-J., Luan Y. (2017). Hydration and Durability of Concrete Containing Supplementary Cementitious Materials. Adv. Mater. Sci. Eng..

[15] Vieira A.P., Toledo Filho R.D., Tavares L.M., Cordeiro G.C. (2021). Mitigation of autogenous shrinkage in high-strength concrete by use of rice husk ashes with distinct porous structures. Adv. Cem. Res..

[16] Ribeiro A.B., Carrajola A., Gonçalves A.F. Effectiveness of Shrinkage-Reducing Admixtures on Different Concrete Mixtures. Proceedings of the SP-217: Seventh CANMET/ACI International Conference on Superplasticizers and Other Chemical Admixtures in Concrete.

[17] Rugytė A., Daukšys M., Juočiūnas S., Borg R. (2020). The Behaviour of Fresh Concrete with Varying Coarse Aggregate Content at the Concrete-Steel Wall Interface. Buildings.

[18] Belie N.D., Soutsos M., Gruyaert E. (2018). Properties of Fresh and Hardened Concrete Containing Supplementary Cementitious Materials: State-of-the-Art Report of the RILEM Technical Committee 238-SCM, Working Group 4.

[19] Craeye B., De Schutter G., Desmet B., Vantomme J., Heirman G., Vandewalle L., Cizer Ö., Aggoun S., Kadri E. (2010). Effect of mineral filler type on autogenous shrinkage of self-compacting concrete. Cem. Concr. Res..

[20] Stock A.F., Hannantt D.J., Williams R.I.T. (1979). The effect of aggregate concentration upon the strength and modulus of elasticity of concrete. Mag. Concr. Res..

[21] Schulze P.D.-I.D. (2008). Powders and Bulk Solids: Behavior, Characterization, Storage and Flow.

[22] Larrard F. (1999). Concrete Mixture Proportioning: A Scientific Approach.

[23] Chidiac S., Maadani O., Razaqpur A.G., Mailvaganam N.P. (2000). Controlling the quality of fresh concrete a new approach. Mag. Concr. Res..

[24] Hidalgo J., Chen C.-T., Struble L.J. (2008). Correlation between paste and concrete flow behavior. Int. Concr. Abstr. Portal.

[25] Wu Q., An X. (2014). Development of a mix design method for SCC based on the rheological characteristics of paste. Constr. Build. Mater..

[26] Hu J., Wang K. (2011). Effect of coarse aggregate characteristics on concrete rheology. Constr. Build. Mater..

[27] Xiao L., Jiang D. (2017). The study of pervious concrete mix proportion by the method of specific surface area of aggregate. Mater. Sci. Eng..

[28] Nazari A., Sanjayan J. (2016). Handbook of Low Carbon Concrete.

[29] Abebe D. (2022). Comparative Analysis of Selected Concrete Mix Design Methods Based on Cost-Effectiveness. Adv. Civ. Eng..

[30] Zhang N., Zuo W., Xu W., Song S.-Y. (2020). A New Approach for Designing Fluid Concrete with Low Cement Content: Optimization of Packing Density of Aggregates. Materials.

[31] Mardani-Aghabaglou A., Kankal M., Nacar S., Felekoğlu B., Ramyar K. (2021). Assessment of cement characteristics affecting rheological properties of cement pastes. Neural Comput. Appl..

[32] Yu B., Yang L., Wu M., Li B. (2014). Practical model for predicting corrosion rate of steel reinforcement in concrete structures. Constr. Build. Mater..

[33] Bellmann F., Damidot D., Möser B., Skibsted J. (2010). Improved evidence for the existence of an intermediate phase during hydration of tricalcium silicate. Cem. Concr. Res..

[34] (2023). Common Portland Cement.

[35] (2014). Test Method for Determinging Cement Density.

[36] (2016). Standard for Test Method of Performance on Ordinary Fresh Concrete.

[37] (2015). Standard Test Method for Slump of Hydraulic-Cement Concrete.

[38] (2019). Standard for Test Methods of Concrete Physical and Mechanical Properties.

[39] (2023). Standard Test Method for Compressive Strength of Cylindrical Concrete Specimens.

[40] Ferraris C.F., Obla K.H., Hill R.L. (2001). The influence of mineral admixtures on the rheology of cement paste and concrete. Cem. Concr. Res..

[41] Roussel N., Stefani C., Leroy R. (2005). From mini-cone test to Abrams cone test: Measurement of cement-based materials yield stress using slump tests. Cem. Concr. Res..

[42] Garboczi E., Bentz D. Percolation Aspects of Cement Paste and Concrete - Properties and Durability, 2000-08-01, 2000. Proceedings of the High-Performance concrete: Research to Practice, ACI Special Publication 189 (SP-189).

[43] Kwan A.K.H., Wong H.H.C. (2007). Packing density of cementitious materials: Part 2—Packing and flow of OPC + PFA + CSF. Mater. Struct..

[44] Wallevik J.E. (2006). Relationship between the Bingham parameters and slump. Cem. Concr. Res..

[45] Popovics S., Ujhelyi J. (2008). Contribution to the Concrete Strength versus Water-Cement Ratio Relationship. J. Mater. Civ. Eng..

[46] Kaplan M.F. (1959). Flexural and Compressive Strength of Concrete as Affected by the Properties of Coarse Aggregates. J. Proc..

[47] Scrivener K.L., Crumbie A.K., Laugesen P. (2004). The Interfacial Transition Zone (ITZ) Between Cement Paste and Aggregate in Concrete. Interface Sci..

[48] Bažant Z.P. (1994). Discussion of “*Fracture Mechanics and Size Effect of Concrete in Tension*” by Tianxi Tang, Surendra, P. Shah, and Chengsheng Ouyang (November, 1992, Vol. 118, No. 11). J. Struct. Eng..

